# Sex-Dimorphism in Aging Gene Regulation: Are We Missing Half of the Picture?

**DOI:** 10.1093/geroni/igaa057.2620

**Published:** 2020-12-16

**Authors:** Bérénice Benayoun, Ryan Lu, Nirmal Sampathkumar, Min Hoo Kim

**Affiliations:** University of Southern California, Los Angeles, California, United States

## Abstract

The existence of human supercentenarians reveals a surprising predictor for exceptional longevity: being female. Not only are 33 out of 34 living supercentenarians women, women are also more resistant to most diseases responsible for age-related morbidity in the US. However, because most molecular aging studies generally opt to use only one sex, sex-driven differences in aging remain poorly understood. A key compartment that can actively respond to sex-specific inputs throughout life is the immune system. Indeed, the majority of age-related diseases share common inflammatory mechanisms, a phenomenon described as “inflamm-aging”. Macrophages play an important role in the inflammatory response throughout life, and are considered major mediators of this phenomenon. Thus, to unbiasedly dissect sex differences in immune aging, we generated ‘omics’ data from 4 and 20 months old female and male mice. Intriguingly, we found that transcriptional aging in primary macrophage populations varies strongly between sexes, with up to 20-fold more aging changes in female vs. male cells. Pathways specifically downregulated in females with aging included lysosome, inflammation and phagolysosome. We confirmed experimentally that metabolic preferences of macrophages are indeed directly modulated in this context (e.g. glycolytic preference for male-derived cells). Our results support the notion that there are functional differences in aging trajectories in the immune system of female vs. male mice. Our research could provide new insights into the molecular underpinnings of sex-dimorphism in aging and disease.

